# Supplementary material for: Engineering of Saccharomyces cerevisiae for 24-Methylene-Cholesterol Production
Source: Biomolecules. 2021 Nov 17;11(11):1710. doi: 10.3390/biom11111710 (PMC8615579; doi:10.3390/biom11111710)
Supplement: Supplementary file 1 [file biomolecules-11-01710-s001.zip › biomolecules-1422908-SI.pdf]

Supplementary Materials

Table S1. Primers used in this study.

Primers Used for the Expression Cassette Construction	
PhDWF5-F	ATGGGGGAGTCTCAGTTGGC
PhDWF5-R	CTAGTAAATTCCGGGTACGACCCTGT
Erg5-UP-F	CCTTTAGTGAGGGTTGAATTCTGGTGTACTTCATTTTCTCAATCAAA
Erg5-UP-R	CACAGCAGCATTGGAATTAGTCAAC
Erg5-PhDWF5-F	CTAATTCCAATGCTGCTGTGTTTTATTTAGGTTCTATCGAGGAGAAA
Erg5-PhDWF5-R	CCGAAGATCAAGTCAGGCAAATTAAAGCCTTCGAGCG
Erg5-OsDWF5-F	CTAATTCCAATGCTGCTGTGTTTTATTTAGGTTCTATCGAGGAGAAA
Erg5-OsDWF5-R	CCGAAGATCAAGTCAGGCAAATTAAAGCCTTCGAGCG
Erg5-XlDWF5-F	CTAATTCCAATGCTGCTGTGTTTTATTTAGGTTCTATCGAGGAGAAA
Erg5-XlDWF5-R	CCGAAGATCAAGTCAGGCAAATTAAAGCCTTCGAGCG
Erg5-DOWN-F	TTGCCTGACTTGATCTTCGGTAATATAGTTACC
Erg5-DOWN-R	AGTGAGTCGTATTACGGATCCAGTTCTGGCCCATCATCGG
Erg4-UP-F	CCTTTAGTGAGGGTTGAATTCTCCACTGCTAATGTACTATATGTGGATT
Erg4-UP-R	CGAAGAGCACCTAGGGTTTAGTGGAGCTCC
Erg4-GENE-F	TAAACCCTAGGTGCTCTTCGCGCGTTTCGGT
Erg4-GENE-R	TCTAACCTCGGTGATGGTTCACGTAGTGGGC
Erg4-DOWN-F	GAACCATCACCGAGGTTAGATTACCTTGGTTCACC
Erg4-DOWN-R	AGTGAGTCGTATTACGGATCCCCATCAATCAATAGGTACGATCCA

Primers Used for the Real-Time Quantitative PCR	
ALG9-F	GTGAACAATTACACAGCTCCTATAG
ALG9-R	CCTATGATTATCTGGCAGCAGGAAAG
qPCR-XL-F	CCTGCTTTGACTTGGACTGCTGT
qPCR-XL-R	GTTCTAGCACCTTCTTGAACACC

Figure S1:

The amino acid sequences of *DHCR7* from *P. angulata*, *O. sativa,* and *X. laevis*:

a. ***P. angulata***

MGESQLAHPPLFTYVSMLTLLTIVPPFIILMWYTNVHADGSILNTFNYLKENGLQGLIDIWP
RPTAVAGKIIICYGLFEAALQILLPGKRVEGPISPTGHRPVYKANGVAAYTVTLITYLSLWW
FGIFNPTIVYDHLGEILSTLNIGSLIFCLLLYIKGHVAPSSTDHGSSGNIIIDFYWGMELYPRIG
KHFDIKVFTNCRFGMISWALLPITYCIKQYEEYGSLSDSMLVHTIITLVYVTKFFWWEAGY
WNTMDIAHDRAGFYICWGCLVFLPCIYTSPGMYLVKHPVNLGPQLALSILAAGILCVYIN
YDCDRQRQEFRRTNGKAPVWGKAPSKIVASYTTTTGETKSSILLTSGWWGLARHFHYVPE
ILASFFWCVPALFNHFIPYFYVVYLTVLLLDRAKRDDERCKAKYGKYWKKYCEKVPYRV
VPGIY

b. ***O. sativa***

MAKPRASAAAAKAPASTPPKTVHSALVTYASMLSLLSLCPPFVILLWYTMVHADGSVVRA
YEHLREHGVLEGLKAIWPMPTMAAWKIIFGFGLFEAALQLLLPGKRFEGPVSPSGNVPVY
KANGLQAYAVTLITYLSLWWFGIFNPAIVYDHLGEIYSALVFGSFVFCIFLYIKGHLAPSSSD
SGSSGNVIIDFYWGMELYPRIGKHFDIKVFTNCRFGMMSWAVLAVTYCIKQYEMNGRVAD
SMLVNTALMLIYVTKFFWWESGYWCTMDIAHDRAGFYICWGCLVWVPSIYTSPGMYLV
NHPVNLGPQLALSILLAGILCIYINYDCDRQRQEFRRTNGKCSIWGKAPSKIVASYQTTNGE
TKSSLLLTSGWWGLSRHFHYVPEILSAFFWTVPALFDHFLPYFYVIFLTILLFDRAKRDDDR
CSSKYGKYWKMYCNKVPCRVIPGIY

c. ***X. laevis***

MGERRRANASRGDKKVANGEKQHVGQWGRAWEVDYFSLASVIFLLAFAPLIVYYFVMS
CDQYQCALTAPVLDLYSGKARLSDIWDKTPALTWTAVKIYLAWVSFQVFLYMFLPDILH
KFVPGYEGGVQEGARTPAGLINKYQVNGLQAWTITHLLWFANAYHFHWFSPTIVIDNWI
PLLWCANLLGYSVATFALVKANFFPTNANDCKFTGNFFYDYMMGIEFNPRIGKWFDFKL
FFNGRPGIVAWTLINLSYAAKQQELYGQVTNSMILVNVLQAIYVVDFFWNESWYLKTIDI
CHDHFGWYLGWGDCVWLPYLYTLQGLYLVYNPVELSTTAAVAVLLLGLIGYYIFRMTN
HQKDLFRRTNGNCKIWGKKPKSIECFYVSADGKRHYSKLMISGFWGVARHLNYTGDLM
GSLAYCLACGFDHLLPYFYFIYMTILLVHRCIRDEHRCSSKYGKDWKLYTSAVPYRLLPG
LF
